# Cefiderocol against Multi-Drug and Extensively Drug-Resistant *Escherichia coli*: An In Vitro Study in Poland

**DOI:** 10.3390/pathogens11121508

**Published:** 2022-12-09

**Authors:** Patrycja Zalas-Więcek, Katarzyna Płachta, Eugenia Gospodarek-Komkowska

**Affiliations:** 1 Department of Microbiology, Ludwik Rydygier Collegium Medicum in Bydgoszcz, Nicolaus Copernicus University (NCU) in Toruń, 85-094 Bydgoszcz, Poland; 2 Clinical Microbiology Division, Dr Antoni Jurasz University Hospital No. 1 in Bydgoszcz, 85-094 Bydgoszcz, Poland

**Keywords:** carbapenemases, cefiderocol, *Escherichia coli*, extended spectrum beta-lactamases, extensively drug-resistance, multi-drug-resistance

## Abstract

Cefiderocol (CFDC) is a novel, broad-spectrum siderophore cephalosporin with potential activity against multi-drug (MDR) and extensively drug-resistant (XDR) Enterobacterales, including carbapenem-resistant strains. We assessed the in vitro susceptibility to CFDC of MDR, and XDR *E. coli* isolates derived from clinical samples of hospitalized patients. Disk diffusion (DD) and MIC (minimum inhibitory concentration) test strip (MTS) methods were used. The results were interpreted based on EUCAST (version 12.0 2022) recommendations. Among all *E. coli* isolates, 98 (94.2%) and 99 (95.2%) were susceptible to CFDC when the DD and MTS methods were used, respectively (MIC range: <0.016–4 µg/mL, MIC_50_: 0.19 µg/mL, MIC_90_: 0.75 µg/mL). With the DD and MTS methods, all (MIC range: 0.016–2 µg/mL, MIC_50_: 0.19 µg/mL, MIC_90_: 0.75 µg/mL) but three (96.6%) ESBL-positive isolates were susceptible to CFDC. Out of all the metallo-beta-lactamase-positive *E. coli* isolates (MIC range: 0.016–4 µg/mL, MIC_50_: 0.5 µg/mL, MIC_90_: 1.5 µg/mL), 16.7% were resistant to CFDC with the DD method, while 11.1% were resistant to CFDC when the MTS method was used. CFDC is a novel therapeutic option against MDR and XDR *E. coli* isolates and is promising in the treatment of carbapenem-resistant *E. coli* strains, also for those carrying Verona integron-encoded metallo-beta-lactamases, when new beta-lactam-beta-lactamase inhibitors cannot be used.

## 1. Introduction

Enterobacterales, including *Escherichia coli*, are important pathogens in hospital and community-acquired infections and can cause many serious infections, such as urinary tract infections, wound infections, intra-abdominal infections, pneumonia, bacteremia, sepsis and neonatal meningitis [1]. Due to the widespread use of antimicrobial agents in clinical treatment, the occurrence of antimicrobial resistance (AMR) of Gram-negative rods has dramatically increased over the past years and now is one of the biggest threats to public health today, both globally and in the WHO (World Health Organization) European Region [2,3]. In 2017 the WHO experts recognized carbapenem-resistant (CR) Enterobacterales, *Pseudomonas aeruginosa* and *Acinetobacter baumannii*, and third-generation cephalosporin-resistant Enterobacterales as ‘priority 1: critical’ pathogens on a global priority list of antibiotic-resistant bacteria. Similarly, the European Center for Disease Prevention and Control (ECDC) raises the alarm on high percentages of resistance to third-generation cephalosporins and carbapenems in Enterobacterales and high percentages of CR *P. aeruginosa* and *Acinetobacter* species [2,3].

Within Enterobacterales rods, *E. coli* is the most frequently isolated pathogen from clinical specimens. Particularly disturbing are multi-drug (MDR) and extensively drug-resistant (XDR), extended-spectrum β-lactamase-producing (ESBL) and/or CR *E. coli* strains due to significant limitations of antimicrobial therapeutic possibilities and thus morbidity and mortality. The prevalence of these kinds of strains has increased over the past years, and this is a serious public health concern worldwide [4]. Resistance of *E. coli* to beta-lactam antibiotics is attributed to the ability to produce ESBLs, mainly CTX (cefotaximase)-M (Munich)-type and carbapenemases: class A (serine carbapenemases, such as *Klebsiella pneumoniae* carbapenemase, KPC), class B (metallo-beta-lactamases, MBL), such as VIM (Verona integron-encoded metallo-beta-lactamase), and NDM (New Delhi metallo-beta-lactamases), as well as class D (oxacillinases, OXA), such as OXA-48-type [4,5]. In Poland, a high prevalence of beta-lactamase-producing and carbapenem-resistant (CR) Gram-negative bacteria is observed. The predominant type of ESBL enzyme is CTX-M-1-group, which is present mainly in *E. coli* and *K. pneumoniae*, whereas the most common carbapenemases are VIM, NDM, and KPC [4,5,6]. In Poland, the phenomenon of resistance to colistin (current the last-line agent) in *E. coli* rods is also a concern, especially if it occurs in CR MDR or XDR *E. coli* isolates [7,8].

The consequences of AMR can be severe, leading to mounting healthcare costs, treatment failure, and death. It is considered that prompt treatment with effective antimicrobials is the most effective way of reducing the risk of poor outcomes from serious infections. Therefore, both the WHO and the ECDC have warned about the shortage of effective antibiotics and urged pharmaceutical companies to develop new drugs [2,9]. Recently, several new antimicrobial agents have been approved for the treatment of Gram-negative rods infections, such as a novel beta-lactams (e.g., ceftazidime/avibactam, ceftolozane/tazobactam, meropenem/vaborbactam, imipenem-cilastatin/relebactam), and a new aminoglycoside (plazomicin) and tetracycline (eravacycline), but unique features of these agents are not able to overcome some resistant mechanisms of Gram-negative rods [9]. None of the novel beta-lactam antibiotics are stable against MBL-producing Gram-negative rods, including *E. coli*. In addition, ESBLs like GES 6 (Guiana-Extended-Spectrum) and PER 1 (*Pseudomonas* extended resistant) confer resistance to ceftolozane-tazobactam, and the KPC-49 variant can confer resistance to ceftazidime/avibactam in *E. coli* strains [10,11].

Cefiderocol (CFDC) (formerly S-649266) is a novel siderophore-conjugated cephalosporin antibiotic developed by Shionogi & Co., Ltd. (Osaka, Japan), with activity against MDR and XDR aerobic Gram-negative rods including Enterobacterales and non-glucose-fermenting rods. This antibiotic has no clinically relevant activity against Gram-positive or anaerobic bacteria due to intrinsic resistance [12].

CFDC (Fetroja^®^, Fetcroja^®^) is an intravenous antibiotic approved in the European Union (EU) and the United States of America (USA) for the treatment of adults with complicated urinary tract infections caused by Enterobacterales and *Pseudomonas aeruginosa* (14 November 2019—the U.S. Food and Drug Administration, FDA), infections caused by aerobic Gram-negative rods with limited treatment options (23 April 2020—The European Medicines Agency (EMA) Committee for Medical Products for Human Use (CMPH)) and hospital-acquired pneumonia and ventilator-associated bacterial pneumonia caused by Enterobacterales, *P. aeruginosa* and *Acinetobacter baumannii* complex. The safety and efficacy of CFDC in children below 18 years of age have not yet been established [13].

CFDC shares a chemical structure in the C-7 side chain with ceftazidime and in the C-3 side chain with cefepime, which enables CFDC to be active against Gram-negative rods and confers stability against beta-lactamases. On the C-3 side chain, CFDC has a catechol moiety that chelates ferric (Fe-III) iron-imitating natural siderophores (Supplementary Figure S1) [12,14]. Because of this molecule, CFDC binds to iron transport channels, and thereby enters the periplasmic space of Gram-negative bacteria, like a ‘Trojan horse’, reaching high concentrations and thus exceeding most bacterial mechanisms, such as efflux pumps, porins and beta-lactamases [15,16]. Once inside, CFDC subsequently binds to penicillin-binding proteins (PBPs): PBP-3 and PBP-2 of the cellular wall, inhibiting bacterial peptidoglycan cell wall synthesis, which leads to cell lysis and death.

The CFDC has potent activity against Enterobacterales producing all four Ambler classes of beta-lactamases, including ESBL, AmpC beta-lactamase and MBL, including VIM and NDM, KPC and OXA [16].

Currently, in Poland, there is no data on the susceptibility of MDR and XDR Gram-negative rods to CFDC. Therefore, the main objective of this study was to evaluate the in vitro susceptibility to CFDC of MDR and XDR *E. coli* isolates derived from clinical specimens of hospitalized patients. CFDC is not available and used in Poland, so this study presents data on the susceptibility to CFDC in MDR and XDR *E. coli* isolates before the use of this antibiotic in our country.

## 2. Materials and Methods

### 2.1. Bacterial Isolates and Identification

The study included 104 non-replicate *E. coli* isolates derived from the collection of the Department of Microbiology Ludwik Rydygier Collegium Medicum in Bydgoszcz, Nicolaus Copernicus University (NCU) in Toruń, Poland. All of them were isolated from April 2016 to September 2022 from clinical specimens of patients hospitalized in different departments of two Polish teaching hospitals. The isolates were derived from different clinical specimens, including blood (14.4%), urine (58.6%), abdominal (5.8%), pleural (1.0%) fluids, stool (4.8%), wound (4.8%), rectal (9.6%) and stoma (1.0%) swabs (Supplementary Table S1). Only one isolate per patient was accepted. All isolates were identified to species by applying mass spectrometry in the MALDI Biotyper system (Bruker) according to the manufacturer’s instructions and selected based on the pulsed-field gel electrophoresis according to the protocol described previously [17].

### 2.2. Phenotypic and Genetic Screening of ESBLs and Carbapenemases

*E. coli* isolates were classified as ESBL-producers based on their resistance to penicillins and extended-spectrum cephalosporins, positive Phoenix M50 ESBL testing (Becton-Dickinson, Franklin Lakes, NJ, USA) and DDST (double-disk synergy test) using the following disks: ceftazidime (30 μg), cefotaxime (30 μg), and amoxicillin/clavulanic acid (20/10 μg) (Oxoid, Hampshire, UK). To increase the sensitivity of the test, disks containing cefepime (30 μg) (Oxoid, Hampshire, UK) were added. In the absence of susceptibility of the strains to at least one of the carbapenems (i.e., imipenem, meropenem or ertapenem), Carba NP test (Bufor B-PER II—Thermo Scientific Waltham, MA, USA; Tienam (imipenem 500 mg + cilastatin 500 mg)—Merck Sharp & Dohme Rahway, NJ, USA; 0.5% Phenol-red solution—Sigma Aldrich St. Louis, MO, USA; ZnSO_4_∙7H_2_O—Merck Sharp & Dohme Rahway, NJ, USA) [18] was performed. To detect the type of carbapenemase, phenotypic tests; i.e., EDTA test for MBL (EDTA—Sigma-Aldrich St. Louis, MO, USA; ceftazidime (30 μg) and imipenem (10 μg)—Oxoid, Hampshire, UK) [19], the boronic acid test for KPC (boronic acid—Sigma-Aldrich St. Louis, MO, USA, meropenem (10 μg)—Oxoid, Hampshire, UK) [20] and 30 µg temocillin test for OXA-48 (Oxoid, Hampshire, UK) [21,22] were applied. Simultaneously with phenotypic tests, ESBL (*bla*_CTX-M-1group_, *bla*_CTX-M-9group_) and carbapenemase (*bla*_KPC_, *bla*_VIM_, *bla*_NDM_, *bla*_OXA-48_, *bla*_OXA-181_) genes were detected using the eazyplex^®^ SuperBug CRE tests (Amplex Biosystems GmbH, Giessen, Germany) based on the loop-mediated isothermal amplification (LAMP) and read out with the help of a Genie II device (Optigene, Horsham, UK), according to the manufacturer’s instruction.

### 2.3. Antimicrobial Susceptibility Testing

*E. coli* isolates were tested for susceptibility to CFDC using disk diffusion (DD) method (30 µg) (Oxoid, Hampshire, UK) and MIC (minimum inhibitory concentration) Test strips (MTS^TM^ Cefiderocol 0.016–256 µg/mL) (Liofilchem, Waltham, MA, USA), using the same standardized inoculum.

Both methods were carried out on standard Mueller-Hinton agar (bio Mèrieux) incubated for 18 ± 2 h at 35 ± 1 °C following the European Committee on Antimicrobial Susceptibility Testing guidelines (EUCAST) [23]. EUCAST (version 12.0 2022) breakpoints of ≥22 mm susceptible, <22 mm resistant and ≤2 µg/mL susceptible, >2 resistant for CFDC, respectively, were used.

Antimicrobial susceptibility testing of other drugs (amoxicillin-clavulanic acid, piperacillin-tazobactam, cefuroxime, cefotaxime, ceftazidime, cefepime, imipenem, meropenem, ertapenem, gentamicin, amikacin, tobramycin, ciprofloxacin, trimethoprim-sulfamethoxazole, tigecycline and colistin) was carried out using NMIC-402 panels that were read out with Phoenix M50 automated system (Becton-Dickinson, Franklin Lakes, NJ, USA) and interpreted according to EUCAST (version 12.0 2022) [23] clinical breakpoints. MDR bacteria were defined as isolates non-susceptible to one or more agents in three or more antimicrobial classes, XDR bacteria as isolates non-susceptible to one or more agents in all but two or fewer classes, and PDR (pan drug resistant) bacteria as non-susceptible to all antimicrobial classes tested [24]. In order to assess the effectiveness of CFDC against *E. coli* strains, on the basis of the MIC values of CFDC obtained for all *E. coli* isolates, the MIC_50_ (MIC required to inhibit the growth of 50% of bacteria) and MIC_90_ (MIC required to inhibit the growth of 90% of bacteria) were determined.

To control the quality of antibiotic susceptibility testing, the standard strains *E. coli* ATCC 25922 and *P. aeruginosa* ATCC 27853 were used according to EUCAST QC (version 12.0 2022) tables [25].

Currently, MTS^TM^ Cefiderocol is validated only for *P. aeruginosa* strains, but due to obtaining the expected quality control results with the reference strains listed above, antimicrobial susceptibility tests for *E. coli* isolates were performed and interpreted (100% inhibition) following the manufacturer’s recommendations for *P. aeruginosa* strains. According to the manufacturer’s technical instructions, using EUCAST breakpoints, the percentage of categorical agreement established by comparison to the broth microdilution (BMD) reference method was stated at 99.3%.

## 3. Results

Out of 104 *E. coli* isolates, 93 (89.4%) and 11 (10.6%) were defined as MDR and XDR, respectively. None of the isolates were PDR. Eighty-nine (85.6%) isolates were ESBL-positive by DDST, Phoenix M50 and eighty-eight (84.6%) by the LAMP method. The LAMP results indicated that 83 (79.8%) and 5 (4.8%) *E. coli* isolates were positive in terms of the *bla*_CTX-M-1 group_ and *bla*_CTX-M-9 group_ genes, respectively. Eighteen (17.3%) isolates produced carbapenemases. Sixteen were MBL-(VIM)-positive, one was MBL-(NDM)-positive, and another one was OXA-48-positive by EDTA test, boronic acid test, respectively, and the LAMP method. The *bla*_VIM_ and *bla*_NDM_ genes were detected in 16 (15.4%), and one of the *E. coli* isolates, respectively. The *bla*_KPC_ and *bla*_OXA-181_ genes were not detected in any of the *E. coli* isolates. Two *E. coli* isolates were positive for both ESBL and carbapenemase genes. One of them was positive for the *bla*_CTX-M-9 group_, and *bla*_OXA-48_ genes, while the second one was positive for the *bla*_CTX-M-1 group_ and *bla*_VIM_ genes.

Among 104 *E. coli* isolates, 98 (94.2%) and 99 (95.2%) were susceptible to CFDC when the DD method and MTS were used, respectively. The diameter range, MIC range, MIC_50_ and MIC_90_ are presented in Table 1.

With the DD and MTS methods, all (MIC range: 0.016–2 µg/mL, MIC_50_: 0.19 µg/mL, MIC_90_: 0.75 µg/mL) but three (96.6%) ESBL-positive isolates were susceptible to CFDC. All ESBL-positive *E. coli* isolates resistant to CFDC were *bla*_CTX-M-1 group_-gene-positive.

Out of all 18 MBL-positive *E. coli* isolates (MIC range: 0.016-4 µg/mL, MIC_50_: 0.5 µg/mL, MIC_90_: 1.5 µg/mL), three (two VIM-positive and one NDM-positive) were resistant to CFDC with the DD method, while two (one VIM-positive and one NDM-positive) were resistant to CFDC when MTS was used.

Out of all tested *E. coli* isolates, six had zone diameter values within the area of technical uncertainty (ATU). The *E. coli* isolates whose susceptible test results were not consistent between the two methods were as follows: first one—was ESBL-positive (*bla*_CTX-M-1 group_-gene-positive) with zone diameter value of 19 mm and MIC value of 3 µg/mL, and the second one—MBL-positive (*bla*_VIM_-gene-positive) with a zone diameter value of 21 mm and a MIC value 0.75 µg/mL. In both cases, the zone diameter values were within ATU.

All six and five *E. coli* isolates resistant to CFDC were also resistant to quinolones and trimethoprim-sulfamethoxazole, respectively.

All (MIC range: 0.016–4 µg/mL, MIC_50_: 0.38 µg/mL, MIC_90_: 2 µg/mL) but three CR *E. coli* isolates were susceptible to CFDC with the MTS method.

## 4. Discussion

CFDC is a novel siderophore cephalosporin with a unique mechanism of bacterial entry. It uses the bacteria’s own system for importing iron to enter the bacterial cell, where it blocks the formation of the bacterial cell wall, killing the bacteria. For this reason, CFDC has broad-spectrum activity against aerobic Gram-negative rods, including MDR and XDR Enterobacterales. CFDC is stable against hydrolysis by beta-lactamases belonging to Ambler Classes A, B, C and D, which gives a potent to be active against carbapenemase-positive Gram-negative rods and is also active against isolates with porin channel mutations or efflux pump mechanism [15,16,26,27].

The main objective of this study was to assess the in vitro susceptibility to CFDC of MDR and XDR *E. coli* isolates, including ESBL-, VIM-, NDM- and OXA-48-positive isolates. All these isolates were derived from clinical samples of hospitalized patients. Out of 104 *E. coli* isolates, 99 (95.2%) were susceptible to CFD, when the MTS was used (MIC range: <0.016–4 µg/mL, MIC_50_: 0.19 µg/mL, MIC_90_: 0.75 µg/mL).

CFDC has been shown to be in vitro active against Gram-negative carbapenemase producers, including those that produce MBLs, such as IMP (imipenemase-producing-metallo-beta-lactamase), NDM, and VIM [16,26,27]. The multinational SIDERO (2014–2016) surveillance studies, in which the subject of research were Gram-negative rods collected in the Asia-Pacific region, Europe and North and South America, showed the broad spectrum of activity of CFDC, with the MIC range 0.004–4 µg/mL against more than 99.0% of all tested Gram-negative isolates [28,29], and more than 97.0% of isolates non-susceptible to carbapenems [30]. For *E. coli*, the MIC_90_ ranged from 0.5 to 1 μg/mL. In our study, CFDC was active in vitro against 90.0% of CR *E. coli* isolates with MIC method, including 15 out of 16 VIM-positive isolates. The NDM-positive *E. coli* isolate was resistant to CFDC. All *E. coli* isolates resistant to CFDC were also resistant to quinolones and trimethoprim-sulfamethoxazole. This may be related to the consumption of antibiotics. Each of the patients from whom the strains were isolated was treated with antibiotics. Three out of six *E. coli* isolates resistant to CFDC by any of the methods were isolated from urine. The patients with suspected urinary tract infections are often started on trimethoprim-sulfamethoxazole and ciprofloxacin. Wong et al. [31] observed high co-resistance rates for ceftriaxone-resistant *E. coli* with ciprofloxacin (73%) and ceftriaxone-resistant *K. pneumoniae* with trimethoprim-sulfamethoxazole (83%), which correlated with consumption of antibiotics. There are currently no data on co-resistance CFDC with quinolones and trimethoprim-sulfamethoxazole in the available literature.

Mechanisms of bacterial resistance that may lead to resistance to CFDC include mutant or acquired PBPs, beta-lactamase enzymes with the ability to hydrolyze CFDC, mutations affecting the regulation of bacterial iron uptake, mutations in siderophore transport proteins and over-expression of native bacterial siderophores [13,32,33,34]. *Wang* et al. [32], in a multicenter study, assessed the susceptibility to CFDC of 181 CR *E. coli* isolates. Among them, 128 (70.7%) of the isolates harbored NDM, 9 (5.0%) harbored KPC and 6 (3.3%) were IMP-positive. CFDC was active against 85.1% of CR *E. coli* isolates (MIC_50_: 2 µg/mL, MIC_90_: 64 µg/mL). All 26 CFDC-resistant *E. coli* produced NDM-5, and one of them also produced KPC-2 carbapenemase. The authors showed that resistance to CFDC of *E. coli* NDM-5-producing is a combination of the premature stop codon of the *cirA* gene (gene for siderophore receptor), *pbp3* gene mutation, and *bla*_NDM-5_ existence. In turn the other authors reported other possible CFDC resistance mechanisms. Kohira et al. [33] indicate an association between beta-lactamase PER (type of ESBL) production and resistance to CFDC, whereas Simner et al. [34] reported an increase in *bla*_NDM_ gene copy number under antibiotic pressure, resulting in high expression of NDM, leading to CFDC resistance. Furthermore, Fröhlich et al. [35] noted that the expression of beta-lactamase genes from various Ambler classes can substantially contribute to CFDC resistance. In the in vitro study, the authors stated that the expression of *bla*_KPC-2_, *bla*_CMY-2_, *bla*_CTX-M-15_ and *bla*_NDM-1_ substantially reduced CFDC susceptibility. Additionally, directed evolution on these enzymes showed that, with the acquisition of only 1–2 non-synonymous mutations, all beta-lactamases were evolvable to further CFDC resistance. However, it is difficult to argue with these reports because, in our study, the mechanisms of CFDC resistance were not investigated.

Moriis et al. [36] assessed the susceptibility to CFDC of 15 CR *E. coli* isolates with the DD (two kinds of disks) and BMD methods. All *E. coli* isolates were susceptible to CFDC with the BMD method, while 80.0% (30 µg HardyDisks—FDA cleared) to 87.0% (30-µg MASTDISCS RUO) were susceptible to CFDC when the DD method was used.

The authors obtained the following MIC results with the BMD method: MIC range: 0.06–2 µg/mL, MIC_50_: 0.25 µg/mL and MIC_90_: 1 µg/mL. On this basis, the authors concluded, that the DD method offers a convenient alternative approach to BMD methods for CFDC antimicrobial susceptibility testing; however, the results of the CFDC susceptibility assessment depend on the type of disks used.

In our study, there were two *E. coli* isolates whose susceptible test results were not consistent between DD and MTS methods. In both cases, the zone diameter values were within ATU. According to the EUCAST [37], laboratories are recommended to start testing CFDC with the disk diffusion method. EUCAST accepted this method as predictive of susceptibility and resistance outside the ATU. At the same time, EUCAST recommends: “Inside the ATU, and as long as there is no alternative method to resolve interpretative uncertainties (e.g., MIC testing in the routine laboratory or assistance from a reference laboratory), ignore the ATU and interpret using the zone diameter breakpoints in the breakpoint table”. For this reason, both of the mentioned *E. coli* isolates were categorized as resistant to CFDC with the DD method. However, when the MTS method was used, 3 µg/mL and 0.75 µg/mL MIC values for first one and the second *E. coli* isolates, were obtained, respectively, which allowed for the categorization of *E. coli* isolates as susceptible to CFDC. This indicates that the use of the disk diffusion method in CFDC susceptibility testing may result in incorrect susceptibility categorization, especially when the diameter of inhibition is within the ATU.

In addition, Moriis et al. [36] observed an interesting phenomenon that the MIC_90_ was higher among non-carbapenemase-producing CR *E. coli* than the carbapenemase-positive isolates. These findings correlate with our results in the context of differences in the susceptibility of CR *E. coli* to CFDC, depending on whether the DD or MIC method is used. However, it is difficult to explain this phenomenon.

Due to the fact that novel beta-lactam antibiotics, such as ceftazidime/avibactam, ceftolozane/tazobactam, imipenem/relebactam, meropenem/vaborbactam are not stable against VIM- and NDM-type carbapenemases, and that in Poland, VIM-type carbapenemase occurs most frequently in *E. coli* strains [4,16], CFDC may prove to be a particularly important antibiotic needed to treat infections caused by VIM-positive *E. coli* strains. At the same time, it should be noted that not all MDR carbapenem-susceptible *E. coli* strains are susceptible to CFDC. In our study, three ESBL-positive isolates susceptible to carbapenems were resistant to CFDC, and all of them were *bla*_CTX-M-1 group_-gene-positive.

The limitation of the study was an objectively small number of the tested MDR and XDR *E. coli* isolates derived from clinical samples of patients hospitalized only in two Polish teaching Hospitals. It is not sufficiently representative at the regional and hospital level. Follow-up studies with a larger and more diverse group of *E. coli* isolates are needed. In particular, studies should be carried out on *E. coli* strains producing NDM and KPC carbapenemases. In addition, the basis of resistance of *E. coli* isolates to CFDC has not been investigated, so these resistance mechanisms require follow-up research.

## 5. Conclusions

CFDC is a novel therapeutic option against MDR and XDR *E. coli* isolates and is promising in the treatment of CR *E. coli* strains and those carrying VIM-type carbapenemases when beta-lactam-beta-lactamase inhibitors cannot be used. Currently, there is no other beta-lactam with activity against these carbapenemase-producing Enterobacterales. Regardless of in vitro susceptibility, CFDC therapy should used with caution, and the decision to use this antibiotic should be made after consultation by a clinical microbiologist with appropriate experience in the management of infectious diseases.

## Figures and Tables

**Table 1:** Antibacterial activity of CFDC against ESBL-positive and CR *E. coli* isolates (*n* = 104).

Resistance Profile (*n*)	CFDC					
	DD Method—Diameter Range (mm)	S	MIC_50_ (µg/mL)	MIC_90_ (µg/mL)	MIC Range (µg/mL)	S
		*n* (%)				*n* (%)
All	13–35	98 (94.2%)	0.19	0.75	<0.016–4	99 (95.2%)
ESBL-positive (89)	16–35	86 (96.6%)	0.19	0.5	0.016–4	86 (96.6%)
CR (20)	13–35	17 (85.0%)	0.38	2	0.016–4	18 (90.0%)
VIM-positive (16)	13–33	14 (87.5%)	0.5	1.5	0.016–4	15 (93.7%)
	**DD method—Zone Diameter (mm)**	**S**	**MIC Value**			**S**
		***n***				***n***
NDM-positive (1)	14	0	4			0
CTX-M-1 and VIM-positive (1)	24	1	0.5			1
CTX-M-9 and OXA-48-positive (1)	35	1	<0.016			1

CFDC—cefiderocol; CTX-M—cefotaximase-Munich; CR—carbapenem-resistant; DD—disk diffusion; ESBL—extended-spectrum beta-lactamase; MIC—minimum inhibitory concentration; MIC_50_—MIC required to inhibit the growth of 50% of bacteria; MIC_90_—MIC required to inhibit the growth of 90% of bacteria; n—number of isolates; NDM—New Delhi metallo-beta-lactamase; S—susceptible; VIM—Verona integron-encoded metallo-beta-lactamase.

## Data Availability

The data presented in this study are available on request from the corresponding author.

## Supplementary Materials

The following supporting information can be downloaded at: https://www.mdpi.com/article/10.3390/pathogens11121508/s1, Figure S1. Chemical structure and important functional groups of cefiderocol (C_30_H_34_ClN_7_O_10_S_2_) [10,12]; Table S1. Characteristics of *E. coli* isolates (*n* = 104).
