# Supplementary material for: Cefiderocol against Multi-Drug and Extensively Drug-Resistant Escherichia coli: An In Vitro Study in Poland
Source: Pathogens. 2022 Dec 9;11(12):1508. doi: 10.3390/pathogens11121508 (PMC9785875; doi:10.3390/pathogens11121508)
Supplement: Supplementary file 1 [file pathogens-11-01508-s001.zip › pathogens-2036659-supplementary.pdf]

pathogens

MDPI

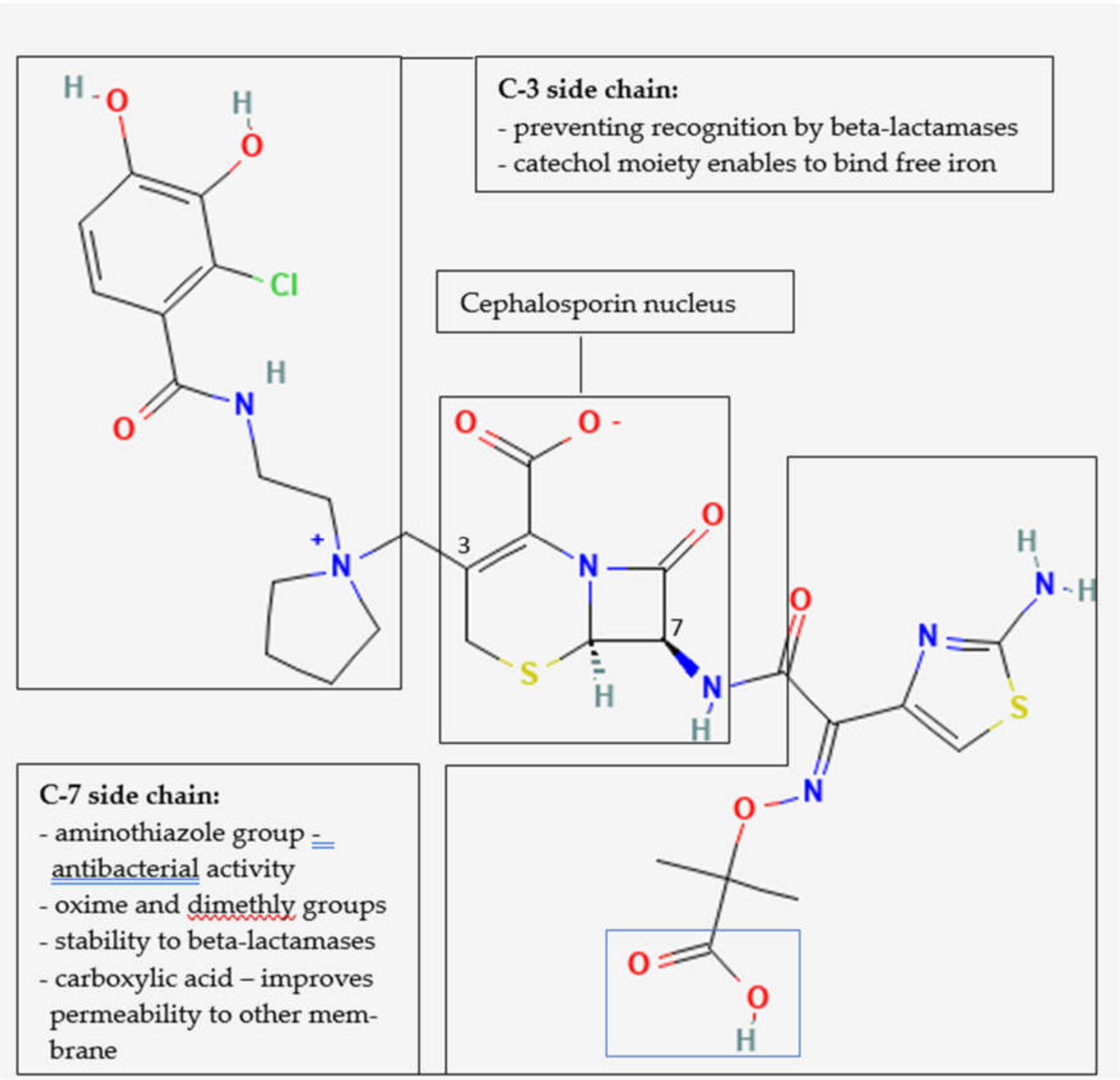

Figure S1. Chemical structure and important functional groups of cefiderocol ($C_{30}H_{34}ClN_7O_{10}S_2$). Based on [10,12].

Table S1. Characteristics of *E. coli* isolates (*n* = 104).

Isolate No.	Collection date (month/year)	Department	Material	Beta-Lactamase Genes	CFDC [mm]	CFDC [SR]	CFDC MIC Value [ug/mL]	CFDC [SR]
34/H1	01/2022	Department of Urology	urine	*bla*CTX-M-1 group	28	S	0.023	S
1081/H1	01/2022	Department of Dermatology	wound swab	*bla*CTX-M-1 group	26	S	0.25	S
2234/H1	01/2017	Department of Nephrology	urine	*bla*CTX-M-1 group	28	S	0.75	S
3526/H1	01/2022	Department of Endocrynology	blood	*bla*CTX-M-1 group	30	S	0.125	S
4746/H2	01/2021	Department of General Surgery	wound swab	*bla*CTX-M-1 group	26	S	0,38	S

5275/H1	01/2022	Department of Cardiology	urine	*bla*CTX-M-1 group	16	R	4	R
5698/H1	02/2017	Department of Urology	blood	*bla*CTX-M-1 group	29	S	0.125	S
5841/H1	02/2017	Department of General Surgery	ab-dominal fluid	*bla*CTX-M-1 group	27	S	0.38	S
7812/H1	01/2022	Anesthesiology and Intensive Care Unit	blood	*bla*CTX-M-1 group	26	S	0.19	S
11283/H1	02/2022	Department of Pediatric Surgery	stoma swab	*bla*CTX-M-1 group	23	S	0.38	S
11898/H1	04/2016	Bone Marrow Transplantation Department	rectal swab	*bla*VIM	24	S	0.5	S
12142/H1	02/2022	Isolation (COVID-19) Department	urine	*bla*CTX-M-1 group	27	S	0.023	S
14134/H1	02/2022	Stroke Department	urine	*bla*CTX-M-1 group	30	S	0.19	S
14255/H1	05/2017	Bone Marrow Transplantation Department	rectal swab	*bla*VIM	28	S	0.064	S
16948/H1	06/2016	Department of Pediatrics, Hematology and Oncology	stool	*bla*CTX-M-1 group	25	S	0.094	S
17583/H1	02/2022	Department of Nephrology	blood	*bla*CTX-M-1 group	29	S	0.19	S
17735/H1	02/2022	Anesthesiology and Intensive Care Unit	blood	*bla*CTX-M-1 group	22	S	0.75	S
18070/H1	06/2016	Department of General Surgery	urine	*bla*CTX-M-1 group	24	S	0.5	S
18092/H1	06/2016	Department of Infectious Diseases and Hepatology	urine	*bla*CTX-M-1 group	32	S	0.032	S
19747/H1	07/2016	Department of Pediatric Surgery	rectal swab	*bla*CTX-M-9 group	30	S	0.094	S
19999/H2	02/2022	Department of Urology	urine	*bla*CTX-M-1 group	31	S	0.19	S
20368/H1	02/2022	Department of Transplantology	urine	*bla*CTX-M-1 group	25	S	0.094	S
22332/H1	08/2016	Neonatal Intensive Care and Neonatal Surgery Unit	rectal swab	*bla*CTX-M-1 group	28	S	<0.016	S
23025/H1	08/2016	Department of Endocrinology	urine	*bla*CTX-M-9 group	27	S	0.047	S
23493/H1	08/2016	Department of Transplantology	urine	*bla*CTX-M-1 group	25	S	0.25	S
23778/H1	08/2016	Department of Urology	blood	*bla*CTX-M-1 group	24	S	0.25	S
23913/H1	08/2016	Department of Nephrology	urine	*bla*CTX-M-1 group	29	S	0.125	S
24633/H1	08/2016	Department of Pediatrics, Hematology and Oncology	urine	*bla*CTX-M-1 group	26	S	0.25	S
25041/H1	03/2022	Department of Urology	urine	*bla*CTX-M-1 group	26	S	0.25	S
25132/H1	09/2016	Anesthesiology and Intensive Care Unit	urine	*bla*CTX-M-1 group	30	S	0.25	S
25177/H1	09/2016	Department of General Surgery	pleural fluid	*bla*CTX-M-1 group	23	S	0.75	S
25211/H1	09/2016	Department of	urine	*bla*CTX-M-1 group	27	S	0.38	S

		Neurology						
25941/H1	09/2017	Department of Transplantology	urine	$bla_{\text{CTX-M-1 group}}$	30	S	<0.016	S
26116/H1	09/2016	Department of Infectious Diseases and Hepatology	blood	$bla_{\text{CTX-M-1 group}}$	27	S	0.032	S
26542/H1	09/2016	Department of Urology	urine	$bla_{\text{CTX-M-1 group}}$	29	S	0.016	S
26644/H1	03/2022	Geriatrics Clinic	urine	$bla_{\text{CTX-M-1 group}}$	25	S	0.064	S
26844/H1	09/2016	Department of Transplantology	urine	$bla_{\text{CTX-M-1 group}}$	27	S	0.125	S
27831/H1	10/2016	Rehabilitation Clinic	urine	$bla_{\text{CTX-M-1 group}}$	23	S	0.38	S
28085/H1	09/2017	Department of General Surgery	ab-dominal fluid	$bla_{\text{CTX-M-9 group}}$	25	S	0.38	S
28667/H1	10/2016	Department of Vascular Surgery	urine	$bla_{\text{CTX-M-1 group}}$	28	S	0.25	S
28694/H1	10/2016	Department of Pediatrics, Hematology and Oncology	stool	$bla_{\text{CTX-M-1 group}}$	30	S	0.38	S
28884/H1	10/2016	Department of Cardiology	urine	$bla_{\text{CTX-M-1 group}}$	32	S	0.125	S
30208/H1	10/2016	Department of Transplantology	urine	$bla_{\text{CTX-M-1 group}}$	29	S	0.38	S
30632/H1	11/2016	Department of Cardiology	urine	$bla_{\text{CTX-M-1 group}}$	27	S	0.19	S
31572/H1	10/2018	Bone Marrow Transplantation Department	stool	bla_{VIM}	25	S	0.75	S
32446/H1	11/2017	Department of Transplantology	wound swab	$bla_{\text{CTX-M-1 group}}$	26	S	0.5	S
40534/H1	12/2018	Department of Cardiology	rectal swab	bla_{VIM}	28	S	0.38	S
46134/H1	07/2022	Department of Pediatrics, Hematology and Oncology	urine	$bla_{\text{CTX-M-1 group}}$	23	S	0.38	S
53314/H1	08/2022	Bone Marrow Transplantation Department	rectal swab	$bla_{\text{CTX-M-1 group}}$	26	S	0.125	S
54037/H1	08/2022	Department of Transplantology	urine	bla_{NDM}	14	R	4	R
58764/H1	07/2021	Department of Cardiology	blood	$bla_{\text{CTX-M-1 group}}$	34	S	<0.016	S
59102/H1	06/2021	Department of Pediatrics, Hematology and Oncology	blood	bla_{VIM}; $bla_{\text{CTX-M-1 group}}$	27	S	0.19	S
59134/H1	06/2021	Department of Urology	urine	$bla_{\text{CTX-M-1 group}}$	29	S	0.047	S
59835/H1	09/2022	Department of Pediatrics, Hematology and Oncology	stool	bla_{VIM}	21	R	0.75	S
81144/H2	01/2022	Department of Cardiology	urine	$bla_{\text{CTX-M-1 group}}$	29	S	0.19	S
81794/H2	01/2022	Department of Hematology	blood	$bla_{\text{CTX-M-1 group}}$	20	R	3	R

82267/H1	10/2021	Department of General Surgery	urine	*bla*$_{CTX-M-1\ group}$	29	S	0.064	S
83937/H1	10/2021	Department of Cardiology	urine	*bla*$_{CTX-M-1\ group}$	29	S	0.016	S
85010/H1	10/2021	Anesthesiology and Intensive Care Unit	blood	*bla*$_{CTX-M-1\ group}$	27	S	0.047	S
85097/H2	01/2022	Department of General Surgery	ab-dominal fluid	*bla*$_{CTX-M-1\ group}$	25	S	0.75	S
88036/H1	10/2021	Department of Urology	urine	*bla*$_{CTX-M-1\ group}$	22	S	0.38	S
88280/H1	10/2021	Department of Trans-plantology	urine	*bla*$_{CTX-M-1\ group}$	28	S	0.125	S
89049/H1	10/2021	Geriatrics Clinic	urine	*bla*$_{CTX-M-1\ group}$	28	S	0.023	S
90249/H2	02/2022	Anesthesiology and Intensive Care Unit	urine	*bla*$_{CTX-M-1\ group}$	28	S	0.19	S
90439/H1	02/2022	Department of Urology	urine	*bla*$_{CTX-M-1\ group}$	19	R	3	S
92779/H1	11/2021	Department of Nephrology	blood	*bla*$_{CTX-M-1\ group}$	29	S	0.094	S
100920/H1	12/2021	Department of Endocrynology	blood	*bla*$_{CTX-M-1\ group}$	31	S	<0.016	S
101984/H1	12/2021	Department of General Surgery	ab-dominal fluid	*bla*$_{CTX-M-1\ group}$	30	S	0.094	S
102135/H1	12/2021	Department of Orthopedics	urine	*bla*$_{CTX-M-1\ group}$	29	S	0.25	S
102240/H1	12/2021	Isolation (COVID-19) Department	urine	*bla*$_{CTX-M-1\ group}$	24	S	0.125	S
102692/H1	12/2021	Geriatrics Clinic	urine	*bla*$_{CTX-M-1\ group}$	24	S	<0.016	S
104118/H1	12/2021	Department of Neurology	urine	*bla*$_{CTX-M-1\ group}$	27	S	0.023	S
112217/H2	02/2020	Department of General Surgery	wound swab	*bla*$_{VIM}$	22	S	1.5	S
247209/H2	04/2018	Department of General Hematology	urine	*bla*$_{VIM}$	25	S	0.38	S
248648/H2	05/2018	Department of General Hematology	rectal swab	*bla*$_{OXA-48}$; *bla*$_{CTX-M-9\ group}$	35	S	<0.016	S
253278/H2	05/2018	Department of Urology	urine	*bla*$_{CTX-M-1\ group}$	25	S	0.25	S
254364/H2	05/2018	Anesthesiology and Intensive Care Unit	urine	*bla*$_{CTX-M-1\ group}$	30	S	0.064	S
256009/H2	05/2018	Department of Cardiology	urine	*bla*$_{CTX-M-1\ group}$	30	S	0.125	S
355159/H2	03/2019	Department of Nephrology	urine	*bla*$_{VIM}$	23	S	1	S
358860/H2	03/2019	Department of General Surgery	ab-dominal fluid	*bla*$_{CTX-M-1\ group}$	30	S	0.125	S
362095/H2	03/2019	Department of Urology	urine	*bla*$_{CTX-M-9\ group}$	31	S	0.094	S
366607/H2	04/2019	Department of Urology	urine	*bla*$_{CTX-M-1\ group}$	25	S	0.5	S
366844/H2	04/2019	Department of Transplantology	urine	*bla*$_{CTX-M-1\ group}$	26	S	0.38	S
368503/H2	04/2019	Anesthesiology and Intensive Care Unit	urine	*bla*$_{CTX-M-1\ group}$	25	S	0.38	S
368329/H2	05/2020	Anesthesiology and Intensive Care Unit	blood	*bla*$_{VIM}$	13	R	4	R
371250/H2	06/2020	Department of Cardiology	urine	*bla*$_{CTX-M-1\ group}$	30	S	0.25	S
583499/H2	08/2020	Department of	stool	*bla*$_{CTX-M-1\ group}$	24	S	0.125	S

		Hematology						
641772/H2	09/2020	Department of Transplantology	urine	*bla*$_{\text{CTX-M-1 group}}$	32	S	0.125	S
652172/H2	09/2020	Department of General Surgery	urine	*bla*$_{\text{CTX-M-1 group}}$	28	S	0.094	S
488423/H2	07/2018	Department of Urology	urine	*bla*$_{\text{VIM}}$	33	S	0.016	S
659534/H2	09/2018	Department of Vascular Surgery	rectal swab	*bla*$_{\text{VIM}}$	27	S	0.19	S
659742/H2	09/2018	Department of Neurology	urine	*bla*$_{\text{CTX-M-1 group}}$	32	S	0.19	S
688208/H2	10/2019	Department of General Surgery	rectal swab	*bla*$_{\text{VIM}}$	23	S	2	S
698738/H2	09/2021	Department of Urology	urine	*bla*$_{\text{CTX-M-1 group}}$	25	S	0.38	S
699186/H2	09/2021	Geriatrics Clinic	urine	*bla*$_{\text{CTX-M-1 group}}$	31	S	0.32	S
719432/H2	10/2018	Department of Nephrology	rectal swab	*bla*$_{\text{VIM}}$	27	S	0.38	S
728539/H2	10/2018	Department of Urology	wound swab	*bla*$_{\text{CTX-M-1 group}}$	31	S	0.125	S
729149/H2	10/2018	Department of General Surgery	ab-dominal fluid	*bla*$_{\text{CTX-M-1 group}}$	22	S	0.75	S
718779/H2	10/2021	Department of General Surgery	blood	*bla*$_{\text{CTX-M-1 group}}$	28	S	0.023	S
718873/H2	10/2021	Department of Urology	urine	*bla*$_{\text{CTX-M-1 group}}$	28	S	1.5	S
729272/H2	11/2021	Department of Cardiol-ogy	urine	*bla*$_{\text{CTX-M-1 group}}$	30	S	0.032	S
729907/H2	11/2021	Department of Transplantology	urine	*bla*$_{\text{CTX-M-1 group}}$	28	S	0.032	S
781123/H2	12/2018	Department of Cardiology	urine	*bla*$_{\text{VIM}}$; *bla*$_{\text{CTX-M-1 group}}$	24	S	0.5	S
899239/H2	12/2019	Department of General Hematology	urine	*bla*$_{\text{VIM}}$	24	S	1	S
E. coli ATCC 25922					28	S	0.064	S
P. aeruginosa ATCC 27853					25	S	0.125	S

MIC - minimum inhibitory concentration; S - susceptible; R – resistant.
